# Revision of the Chinese species of *Dialineura* Rondani, 1856 (Diptera, Therevidae, Therevinae)

**DOI:** 10.3897/zookeys.235.3854

**Published:** 2012-10-31

**Authors:** Si-Pei Liu, Ding Yang

**Affiliations:** 1 Department of Entomology, China Agricultural University, Beijing 100193, China

**Keywords:** Therevidae, *Dialineura*, new species, key to the world, distribution, China

## Abstract

The genus *Dialineura* Rondani is reviewed from China. One species, *Dialineura elongata* **sp. n.** is described as new to science. Two species, *Dialineura nigrofemorata* Kröber and *Dialineura gorodkovi* Zaitzev, are recorded from China for the first time. The female information of *Dialineura henanensis* Yang is also included here. A key to all the male species in the world and a biogeography map of China are presented.

## Introduction

The genus *Dialineura* Rondani, 1856 belongs to the subfamily Therevinae (Diptera: Therevidae). Until now, this genus had twelve known species in the world, which are distributed in Palaearctic region, Oriental region (i.e. China) and Nearctic region (i.e. Canada and USA). Four species have been known to occur in China: *Dialineura kikowensis* Ôuchi, 1943 from Zhejiang; *Dialineura affinis* Lyneborg, 1968 from Sichuan; *Dialineura aurata* Zaitzev, 1971 from Northeast China; *Dialineura henanensis* Yang, 1999 from Henan.

Both Irwin and Lyneborg (1981a) and Webb and Irwin (1991) gave the detailed descriptions for body and male terminalia. Irwin and Lyneborg (1981a) emphasized mid coxa with long pile on posterior surface, and Webb and Irwin (1991) presented the description and figures for female terminalia. The genus *Dialineura* can be characterized by the following features: male eyes nearly contiguous medially; male and female frons pruinose; parafacial usually bare; scape of antenna more or less swollen, wider than first flagellomere; style one-segmented, terminal spine minute; prosternal furrow pilose; scutal chaetotaxy (pairs): *np* 3–6, sa 2, pa 1–2, dc 1–3, sc 1–2; cell m_3_ open; middle coxa with long pile on posterior surface; hind femur with 6–10 anteroventral setae; in male genitalia, hypandrium absent; some species with substylus on gonocoxite; in female genitalia, spermathecal sac duct very short (Irwin and Lyneborg 1981a; Nagatomi and Lyneborg 1988; Webb and Irwin 1991).

## Material and methods

The specimens were studied and illustrated with an OPTEC SMZ-B_2_ stereomicroscope. Male genitalic preparations were made by removing and heating the apical portion of the abdomen in lactic acid at 180°C temperature for 15 min, and rinsing in water and 75% ethanol. Female genitalic preparations were made by removing and rinsing the abdomen in a saturated NaOH solution at room temperature for one day, staining with a saturated Chlorazol Black solution in 75% ethanol, and rinsing in 75% ethanol. After examination, preparations were transferred to fresh glycerin and stored in a microvial pinned below the specimen. The habitus photographs of adults were taken with a digital camera (Canon EOS 450D). Type specimens were deposited in the Entomology Museum of China Agricultural University (CAU), Beijing.

Morphological terminology follows Winterton et al. (1999). The term antennal ratio is defined as length ratio of scape : pedicel : first flagellomere : style. The term substylus is according to Zaitzev (1971), Nagatomi and Lyneborg (1988) and Webb and Irwin (1991), describing a medial spinous process of gonocoxite.

The following abbreviations are used:

## Taxonomy

**Figures 1–6.**
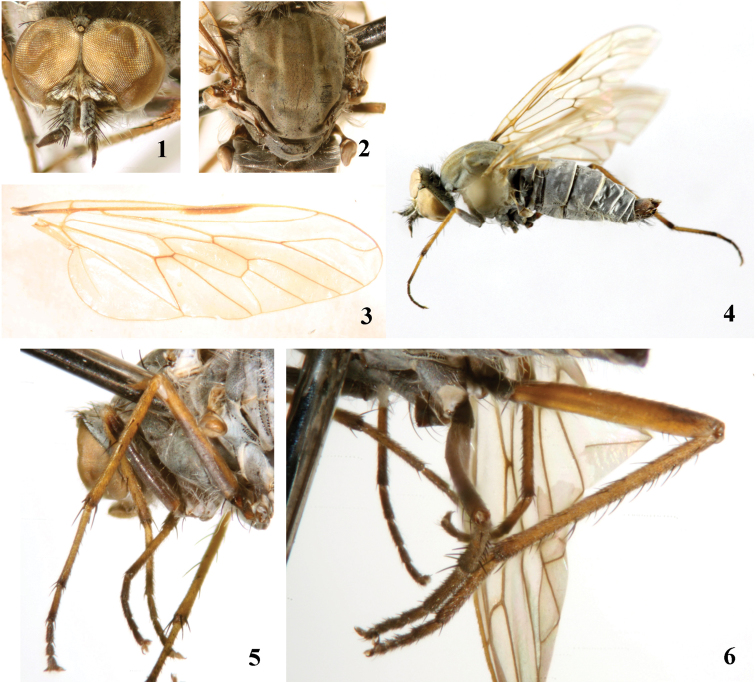
*Dialineura elongata* **sp. n.** Male. **1** head, frontal view **2** mesonotum **3** wing **4** habitus of male, lateral view **5** mid leg **6** hind leg.

Following is a key to the world male species of the genus *Dialineura*. The males of *Dialinuera kikowensis* Ôuchi, 1943 and *Dialineura aurata* Zaitzev, 1971 are unknown, but we can identify females of these two species according to their distinct characters. The female *Dialineura kikowensis* has two wide yellow vittae on mesonotum (Fig. 71), apical margin of cell *m_3_* narrower than cross-vein m-cu (see fig. 1, Ôuchi 1943), each tergites 2–7 with a median black spot (Fig. 71); female *Dialineura aurata* has the body completely covered with dense bright yellow pubescence (Fig. 72).

Some figures cited in the key for the previously described species are from Zaitzev (1971, 1977), Nagatomi and Lyneborg (1988) and Webb and Irwin (1991).

**Table:** 

1	Substylus on gonocoxite absent	2
–	Substylus on gonocoxite present (Figs 52, 66)	6
2	Frons entirely with white pile	*Dialineura albata* (Coquillett, 1898)
–	Frons with some black setae	3
3	Distiphallus basally stout and ventrally curved (see Figs 2, 3, Zaitzev 1971	4
–	Distiphallus basally slender and recurved (Figs 14, 34	5
4	Dorsal apodeme of aedeagus distinctly raised in lateral view, distiphallus wide in dorsal view (see fig. 2, Zaitzev 1971)	*Dialienura anilis* (Linné, 1761)
–	Dorsal apodeme of aedeagus relatively smooth in lateral view, distiphallus tapering in dorsal view (see fig.3, Zaitzev 1971)	*Dialienura mongolica* Zaitzev, 1971
5	Femora black with yellow tips (Fig. 26), pterostigma of wing pale yellow (Fig. 25); apical epandrium narrow with a triangular medial invagination (Fig. 30), dorsal apodeme of aedeagus 1/2 as long as ventral apodeme (Fig. 34)	*Dialineura henanensis* Yang, 1999
–	Mid and hind femora mostly yellow (Figs 5, 6), pterostigma of wing brown (Fig. 3); apical epandrium wide with a trapezoidal medial invagination (Fig. 10), dorsal apodeme of aedeagus nearly as long as ventral apodeme (Fig. 14)	*Dialineura elongata* sp. n.
6	Distiphallus serrated at lateral edges (Fig. 67, 68 and see fig. 19, Lyneborg 1968	*Dialineura affinis* Lyneborg, 1968
–	Distiphallus relatively smooth at lateral edges	7
7	Scape of antenna very large, at least 1.5 times longer than first flagellomere (see fig. 7, Zaitzev 1977; and fig. 15, Nagatomi and Lyneborg 1988)	8
–	Scape of antenna at most 1.3 times longer than first flagellomere	9
8	Tergites 2–4 with a black basal spot; subepandrial sclerite triangular (see fig. 5, Zaitzev 1977), dorsal apodeme of aedeagus shorter than 2/3 length of ejaculatory apodeme (see fig. 4, Zaitzev 1977)	*Dialineura lehri* Zaitzev, 1977
–	Tergites 2–6 with a black basal band which produced medially; subepandrial sclerite trapezoidal (see fig. 28, Nagatomi and Lyneborg 1988), dorsal apodeme of aedeagus nearly as long as ejaculatory apodeme (see fig. 24, Nagatomi and Lyneborg 1988)	*Dialineura shozii* Nagatomi & Lyneborg, 1988
9	Subepandiral sclerite triangular, at most 2 times longer than cercus (see fig. 5, Webb and Irwin 1991)	*Dialineura gorodkovi* Zaitzev, 1971
–	Subepandrial sclerite constricted medially, at least 3 times longer than cercus (Fig. 51 and see figs 7, 8 and 10, Zaitzev 1971)	10
10	Only white pile present on fore femur (Fig. 45); epandrium 2 times longer than subepandrial sclerite (Fig. 51 and see fig. 6, Zaitzev 1971)	*Dialineura nigrofemorata* Kröber, 1937
–	Black pile distinctly present on fore femur; epandrium 1.5 times longer than subepandrial sclerite (see fig. 9, Zaitzev 1971)	*Dialineura lyneborgi* Zaitzev, 1971

### Dialineura elongata sp. n.

urn:lsid:zoobank.org:act:76D30027-1357-4ED4-96A7-148FDC879EB1

http://species-id.net/wiki/Dialineura_elongata

Figs 1 [Fig F2] [Fig F3] 22 73

#### Diagnosis.

Male mesonotum with 3 wide brown vittae, separated by 2 narrow pale yellow stripes, the central vitta with a narrow grey stripe in the middle; female mesonotum with 3 wide brown vittae, separated by 2 narrow pale brown stripes, the central vitta with a narrow pale brown to dark brown stripe in the middle. Mid and hind femora mostly yellow. Pterostigma of wing brown. Male apical epandrium relatively wide with a trapezoid medial invagination; gonocoxite relatively wide apically; dorsal apodeme of aedeagus nearly as long as ventral apodeme; distiphallus recurved and S-shaped.

#### Description.

Male. Body length 7.1–8.5 mm, wing length 6.0–7.0 mm.

Head (Fig. 1) with dense pale pubescence over black ground color, central area of frons brown. White pile from gena to occiput, black setae on ocellar tubercle and frons, parafacial bare, upper occiput also with some black postocular setae. Eyes reddish brown and nearly contiguous on upper frons. Antenna with dense pale pubescence over black ground color, except first flagellomere and style brown; black setae on scape long and thick, but those on pedicel short and thin; first flagellomere nearly bare; central part of first flagellomere widest; style resting apically on first flagellomere with a tiny distal spine; antennal ratio: 5.0 : 1.0 : 4.1 : 0.7. Proboscis pale yellow with some black parts marginally, covered with short brown pile; palpus pale yellow with white pile.

Thorax with dense pale pubescence over black ground color; mesonotum (Fig. 2) with 3 wide brown vittae, separated by 2 narrow pale yellow stripes, the central vitta with a narrow grey stripe in the middle. Notum with sparse short white pile mixed with short black pile marginally, prosternum and pleuron with white pile; macrosetae on thorax black. Scutal chaetotaxy (pairs): *np* 3, *sa* 2, *pa* 1, *dc* 2, *sc* 2. Coxae and trochanters pale pollinose over black ground color, fore femur with pale pubescence over black ground color except apex yellow, both mid and hind femora (Figs 5, 6) yellow, except ventral surface of mid femur and apical dorsal part of hind femur dark brown, tibiae brownish yellow with dark brown apices, all tarsomeres 1–2 brownish yellow with dark brown apices, basal part of hind tarsomere 3 brownish yellow but apical part dark brown, other tarsomeres dark brown. White pile present on coxae and femora, setae on legs black. Fore coxa with *a* 1–2, *av* 1; mid coxa with *a* 3; hind coxa with *a* 2–3, *d* 1. Fore and mid femora without any prominent setae; hind femur with *av* 6, *pv* 3. Fore tibia with *ad* 2–4, *pd* 2–5, *pv* 4, apically with 7 setae; mid tibia with *ad* 3–4, *pd* 3, *av* 2–3, *pv* 2–5, apically with 6 setae; hind tibia with *ad* 5–8, *pd* 5–8, *av* 5–8, *pv* 4–7, apically with 6 setae. Wing (Fig. 3) hyaline tinged yellow; pterostigma very narrow, brown, at end of R_1_; veins brown. Halter stalk pale yellow but dark brown basally and apically; knob brown.

Abdomen with dense pale pubescence over black ground color, posterior margin of each segment pale yellow. White pile on abdomen, some black setae on terminalia. **Male genitalia:** Epandrium (Fig. 10) elongated, 1.4 times longer than wide, apically with a trapezoidal medial invagination. Subepandrial sclerite rectangular, as long as cercus. Gonocoxite (Fig. 11) relatively wide apically. Dorsal apodeme of aedeagus (Figs 12–14) nearly as long as ventral apodeme; distiphallus recurved and S-shaped.

Female. Body length 8.9–10.5 mm, wing length 6.5–7.9 mm.

Most characters of female are similar to the male, with following exceptions: Frons (Fig. 15) with dense dark brown pubescence over black ground color. Frons wide with 2 rows black setae, the narrowest point of frons 5 times wider than anterior ocellus. Antenna ratio: 4.2 : 1.0 : 3.9 : 0.7. Proboscis black but pale yellow marginally. Mesonotum (Fig. 16) with 3 wide brown vittae, separated by 2 narrow pale brown stripes, the central vitta with a narrow pale brown to dark brown stripe in the middle. Fore coxa with *a* 1, *av* 1; mid coxa with *a* 3; hind coxa with *a* 3, *d* 1. Fore and mid femora without any prominent setae; hind femur with *av* 6, *pv* 2. Fore tibia with *ad* 4, *pd* 4, *pv* 4, apically with 5 setae; mid tibia with *ad* 3–4, *av* 5, *pd* 4–5, *pv* 5, apically with 5 setae; hind tibia with *ad* 8, *pd* 9–10, *av* 8, *pv* 9, apically with 3 setae. Pale pubescence on abdomen thinner than the male. **Female genitalia:** Tergite 8 (Fig. 19) slightly longer than wide in dorsal view; sternite 8 (Fig. 20) rectangular in ventral view with an incision apically. Cercus (Fig. 22) semicircular. Subepandrial sclerite (Fig. 20) bell-shaped. Furca (Fig. 21) 1.7 times longer than wide. Accessory glands with separated ducts. Spermathecal sac rather large and spherical; two spermathecae.

#### Type material.

Holotype male, **CHINA:** Shaanxi, Zhouzhi, Houzhenzi (33°53’N, 108°02’E), 1. V. 2009, Mao-Ling Sheng. Paratypes: 3 male, same data as holotype; 1 male, 2 female, **CHINA:** Yunnan, Xishuangbanna, Jinghong (21°58’N, 100°48’E, 300m), 27. IV. 2002, Wen-Quan Zhen; 1 male, **CHINA:** Shaanxi, Zhouzhi, Houzhenzi (33°53’N, 108°02’E), 8. V. 2009, Mao-Ling Sheng; 1 male, 1 female, **CHINA:** Beijing, Botanical Garden (39°59’N, 116°12’E), 24. IV. 2006, Hui Dong.

#### Distribution.

**Palaearctic region: China (Shaanxi, Beijiia:** Tergite 8 (Fig. 19) slightly longer than wide in dorsal view; sternite 8 (Fig. 20) rectangular in ventral view with an incision apically. Cercus (Fig. 22) semicircular. Subepandrial sclerite (Fig. 20) bell-shaped. Furca (Fig. 21) 1.7 times longer than wide. Accessory glands with separated ducts. Spermathecal sac rather large and spherical; two spermathecae.

#### Type material.

Holotype male, **CHINA:** Shaanxi, Zhouzhi, Houzhenzi (33°53’N, 108°02’E), 1. V. 2009, Mao-Ling Sheng. Paratypes: 3 male, same data as holotype; 1 male, 2 female, **CHINA:** Yunnan, Xishuangbanna, Jinghong (21°58’N, 100°48’E, 300m), 27. IV. 2002, Wen-Quan Zhen; 1 male, **CHINA:** Shaanxi, Zhouzhi, Houzhenzi (33°53’N, 108°02’E), 8. V. 2009, Mao-Ling Sheng; 1 male, 1 female, **CHINA:** Beijing, Botanical Garden (39°59’N, 116°12’E), 24. IV. 2006, Hui Dong.

#### Distribution.

Palaearctic region: China (Shaanxi, Beijing); Oriental region: China (Yunnan) (Fig. 73). This is biogeographically part of North China Region and South China Region (Zhang 1999).

#### Remarks.

This new species is similar to *Dialineura henanensis* Yang from China, especially in the recurved and S-shaped distiphallus and the relatively wide apical gonocoxite. But it can be separated from the following features: most areas of the mid and hind femora of both male and female are yellow; the pterostigma of the wing is brown; the halter knob is brown; the epandrium is wide apically with a trapezoidal medial invagination; the subepandrial sclerite is rectangular, as long as the cercus; the dorsal apodeme of aedeagus is nearly as long as the ventral apodeme. In *Dialineura henanensis*, most areas of the mid and hind femora are black; the pterostigma of the wing is pale yellow; the halter knob is pale yellow; the epandrium is narrow apically with a triangular medial invagination; the subepandrial sclerite is triangular, nearly 2 times longer than the cercus; the dorsal apodeme of aedeagus is 1/2 as long as the ventral apodeme.

#### Etymology. 

The specific name refers to the elongated distiphallus, from the Latin adjective “elongatus” meaning prolonged.

**Figures 7–14. F2:**
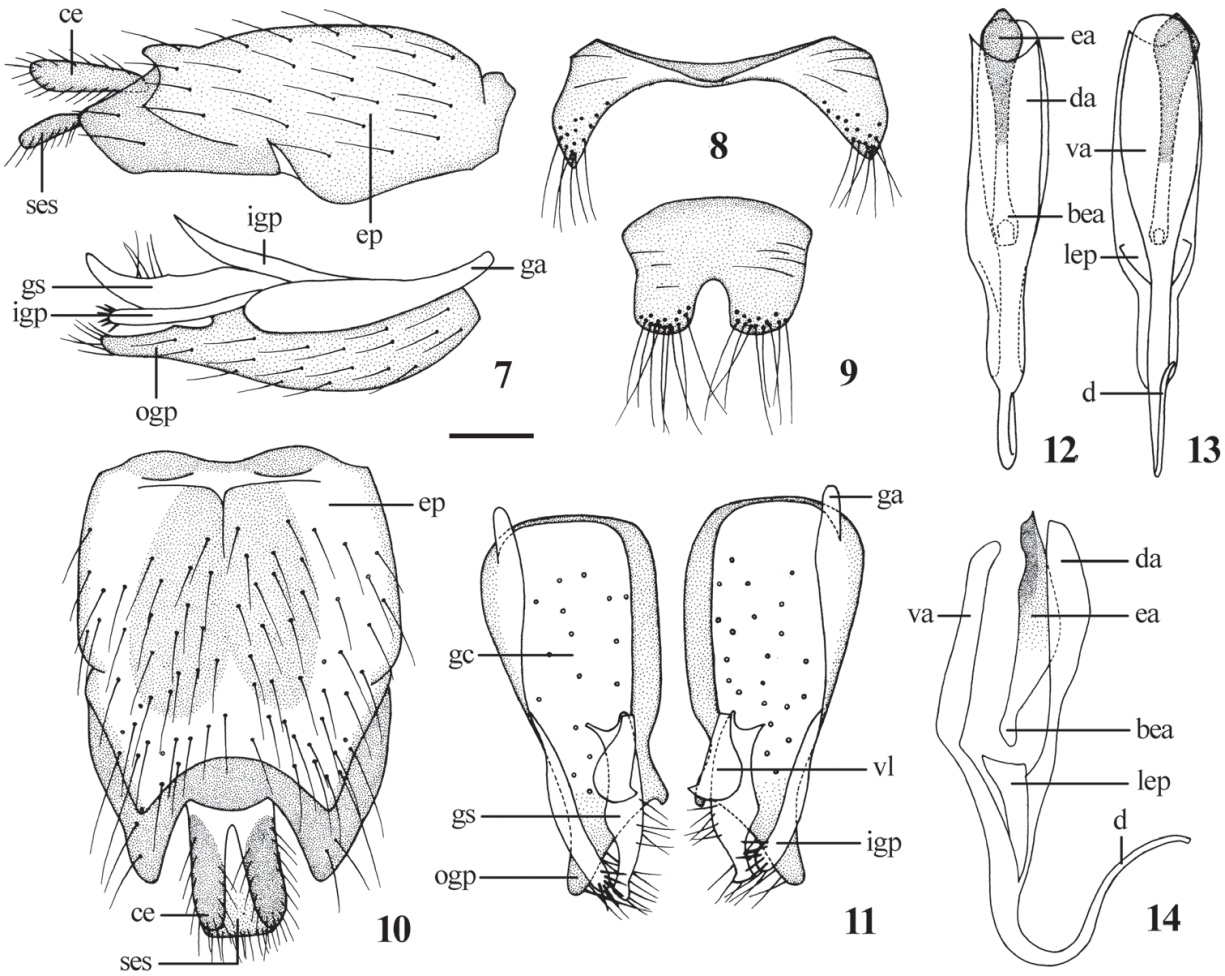
*Dialineura elongata* **sp. n.** Male. **7** terminalia, lateral view **8** tergite 8 **9** sternite 8 **10** epandrium, cercus and subepandrial sclerite, dorsal view **11** gonocoxite and gonostylus, dorsal view **12** aedeagus, dorsal view **13** aedeagus, ventral view **14** aedeagus, lateral view. Scale: 0.2 mm.

**Figures 15–18. F3:**
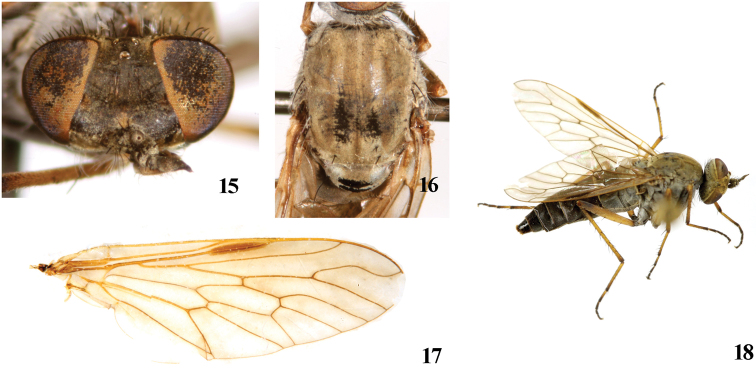
*Dialineura elongata* **sp. n.** Female. **15** head, frontal view **16** mesonotum **17** wing **18** habitus of female, lateral view.

**Figures 19–22.**
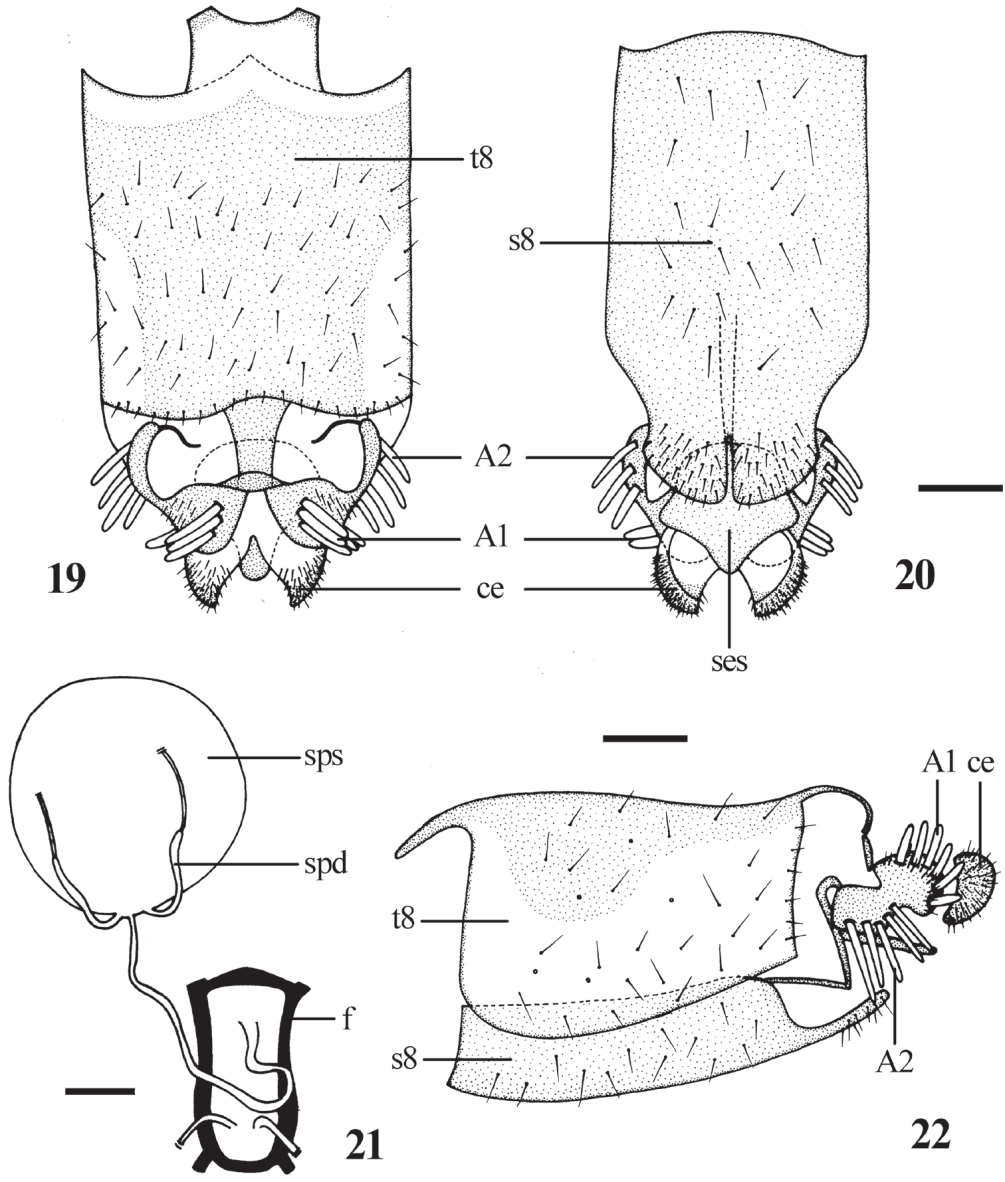
*Dialineura elongata* **sp. n.** Female. **19** terminalia, dorsal view **20** terminalia, ventral view **21** internal reproductive organs **22** terminalia, lateral view. Scale: 0.2 mm.

### Dialineura henanensis

Yang, 1999

http://species-id.net/wiki/Dialineura_henanensis

**Figures 23–26.**
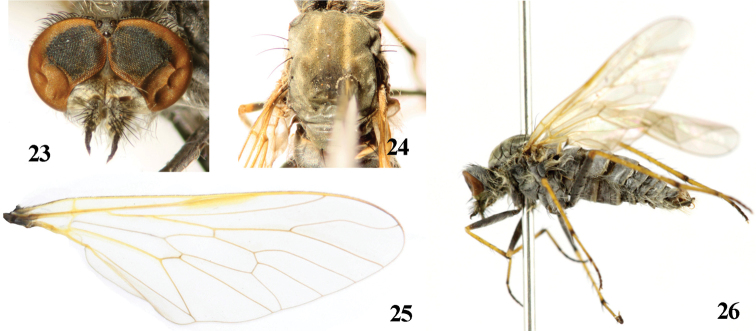
*Dialineura henanensis* Yang. Male. **23** head, frontal view **24** mesonotum **25** wing **26** habitus of male, lateral view.

Figs 23 [Fig F6] [Fig F7] 42 73

Dialineura henanensis Yang, 1999: 186. Type locality: Henan, China (Holotype deposited in Entomology Museum of China Agricultural University, Beijing).

#### Diagnosis.

Male mesonotum with 3 wide grey vittae, separated by 2 narrow pale yellow stripes, the central vitta with a narrow brown stripe in the middle; female mesonotum with 3 wide black vittae, separated by 2 narrow grey stripes, the central vitta with a narrow grey stripe in the middle. Pterostigma of wing pale yellow. Halter knob pale yellow. Male epandrium wide basally then suddenly narrow after middle line and with a triangular medial invagination apically; gonocoxite relatively wide apically; dorsal apodeme of aedeagus 1/2 as long as ventral apodeme; distiphallus recurved and S-shaped.

#### Redescription.

Male. Body length 7.3–8.5 mm, wing length 6.6–7.1 mm.

Other characters as described for *Dialineura elongata* sp. n., with following exceptions: Antennal ratio: 5.5 : 1.0 : 4.5 : 0.5. Proboscis black with short white pile; palpus black with white pile. Mesonotum (Fig. 24) with 3 wide grey vittae, separated by 2 narrow pale yellow stripes, the central vitta with a narrow brown stripe in the middle. Notum with sparse short white pile mixed with black pile. All femora (Fig. 26) black with yellow tips. Fore coxa with *a* 1, *av* 1; mid coxa with *a* 3; hind coxa with *a* 2–3, *d* 1. Fore and mid femora without any prominent setae; hind femur with *av* 6, *pv* 2–3. Fore tibia with *ad* 3–4, *pd* 3, *pv* 3, apically with 4 setae; mid tibia with *ad* 3, *pd* 3, *av* 3, *pv* 4, apically with 6 setae; hind tibia with *ad* 9, *pd* 6–8, *av* 8, *pv* 4–5, apically with 8 setae. Pterostigma of wing (Fig. 25) pale yellow; veins yellow. Halter stalk brownish yellow basally and dark brown apically; knob pale yellow. Anterior margins of tergites 2–3 of abdomen with very thin pubescence so that ground color is visible. Terminalia with only white pile. **Male genitalia:** Epandrium (Fig. 30) much elongated, 1.3 times longer than wide, apically narrowed with a triangular medial invagination. Subepandrial sclerite triangular, nearly 2 times longer than cercus. Dorsal apodeme of aedeagus (Figs 32–34) 1/2 as long as ventral apodeme.

Female. Body length 9.3–10.1 mm, wing length 7.0–7.9 mm.

Most characters of female are similar to the male, with following exceptions: Frons and antenna with dense brownish yellow pubescence over black ground color. Frons (Fig. 35) wide with 2 rows black setae, the narrowest point of frons 3–5 times wider than anterior ocellus. Antennal setae shorter than male; antennal ratio: 4.5 : 1.0 : 4.1 : 0.6. Proboscis with short brown pile. Mesonotum (Fig. 36) with 3 wide black vittae, separated by 2 narrow grey stripes, the central vitta with a narrow grey stripe in the middle. Notum with more pile than male. Fore coxa with *a* 1, *av* 1; mid coxa with *a* 3; hind coxa with *a* 3, *d* 1. Fore and mid femora without any prominent setae; hind femur with *av* 5–7, *pv* 4–5. Fore tibia with *ad* 3, *pd* 3, *pv* 2–4, apically with 6 setae; mid tibia with *ad* 3, *pd* 3, *av* 2, *pv* 4, apically with 6–8 setae; hind tibia with *ad* 7–9, *pd* 10, *av* 8, *pv* 5–7, apically with 6 setae. Most anterior margins of tergites of abdomen with very thin pubescence so that ground color is visible. White short setae mixed with brown short setae on abdomen, except tergite 1 with some white pile. **Female** **genitalia:** Tergite 8 (Fig. 39) quadrate in dorsal view; sternite 8 (Fig. 40) long trapezoidal in ventral view with an incision apically. Cercus (Fig. 42) semicircular. Subepandrial sclerite (Fig. 40) bell-shaped. Furca (Fig. 41) 2.2 times longer than wide. Accessory glands with separated ducts. Spermathecal sac rather large and spherical; two spermathecae, spherical.

#### Materials.

1 male, **CHINA:** Beijing, Shangfang Mountain (39°39’N, 115°49’E), 22. V. 1976, Chi-Kun Yang; 4 male, 14 female, **CHINA:** Yunnan, Xishuangbanna, Jinghong (21°58'N, 100°48'E, 300m), 27. IV. 2002, Wen-Quan Zhen; 2 male, **CHINA:** Henan, Neixiang, Baotianman (33°31'N, 111°52'E), 20. V. 2006, Wei-Hai Li; 3 male, 3 female, **CHINA:** Beijing, Mentougou (39°56'N, 116°06'E), 30. V. 2008, Tao Wang; 1 male, **CHINA:** Shaanxi, Zhouzhi, Houzhenzi (33°53'N, 108°02'E), 5. V. 2009, Mao-Ling Sheng; 1 male, 1 female, **CHINA:** Yanqing, Song Mountain (40°29'N, 115°49'E; 780 m), 23. V. 2009, Wei-Na Cui & Jin-Jing Wang; 10 male, 22 female, **CHINA:** Beijing, Xiaolongmen Woodland (39°57'N, 115°26'E), 24. V. 2009, Li Shi, Hui Yu & Liang Liang; 52 male, 76 female, **CHINA:** Beijing, Xiaolongmen Woodland (39°57'N, 115°26'E; 1 177–1 430 m), 25. V. 2009, Li Shi, Hui Yu & Liang Liang; 16 male, 17 female, **CHINA:** Beijing, Ling Mountain, Ancient Road (39°59'N, 115°29'E; 1 022–1 144 m); Li Shi & Liang Liang; 1 female, **CHINA:** Beijing, Xiaolongmen (39°57'N, 115°26'E), 21. V. 2010, Tao Li; 1 male, **CHINA:** Inner Mongolia, Helan Mountain, Gulaben, Luanchaigou (39°00'N, 105°50'E), 10. VIII. 2010, Li-Hua Wang.

#### Distribution.

Palaearctic region: China (Henan, Beijing, Shaanxi, Inner Mongolia); Oriental region: China (Henan, Yunnan) (Fig. 73). This is biogeographically part of North China Region, Mongolia-Xinjiang Region and South China Region (Zhang 1999).

#### Remarks.

Yang (1999) first described *Dialineura henanensis* from Henan, China and gave the figures of male genitalia, but the female was unknown. We found large amount of *Dialineura henanensis* specimens from other provinces (i.e. Shaanxi, Inner Mongolia, Yunnan) and city (i.e. Beijing) of China, therefore we infer that this species is widespread in China. We redescribe this species and give figures of both male and female genitalia.

**Figures 27–34. F6:**
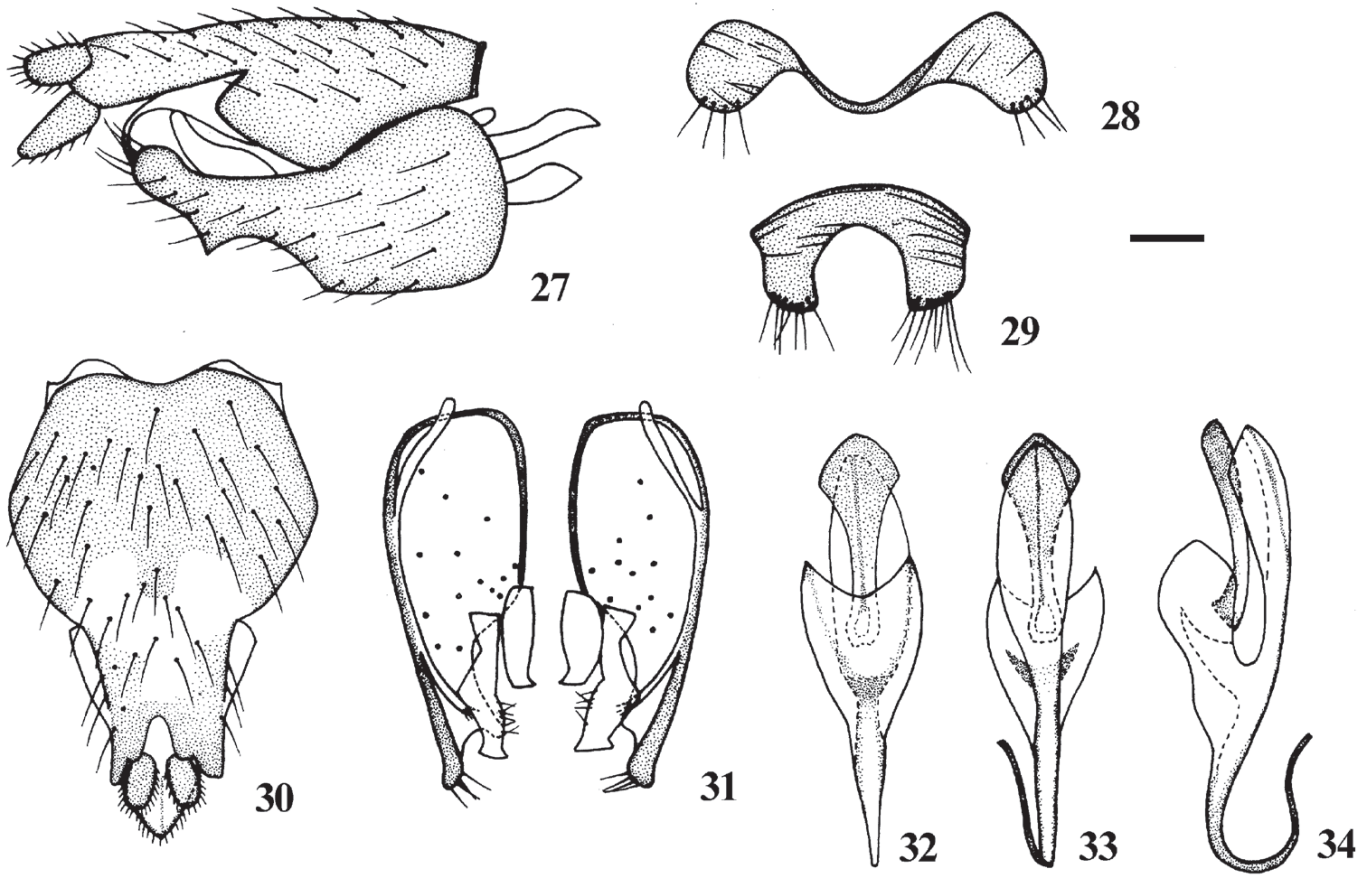
*Dialineura henanensis* Yang. Male. **27** terminalia, lateral view **28** tergite 8 **29** sternite 8 **30** epandrium, cercus and subepandrial sclerite, dorsal view **31** gonocoxite and gonostylus, dorsal view **32** aedeagus, dorsal view **33** aedeagus, ventral view **34** aedeagus, lateral view. Scale: 0.2 mm.

**Figures 35–38. F7:**
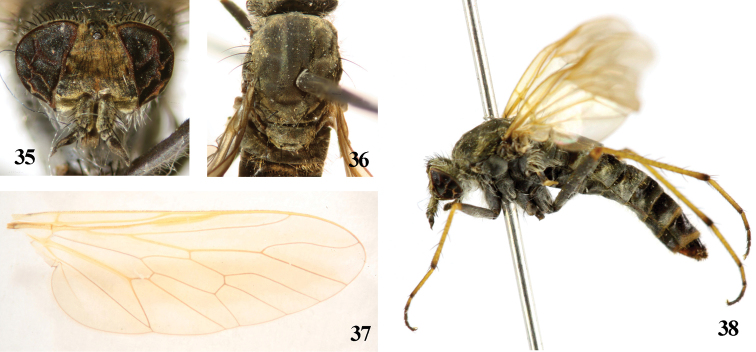
*Dialineura henanensis* Yang. Female. **35** head, frontal view **36** mesonotum **37** wing **38** habitus of female, lateral view.

**Figures 39–42.**
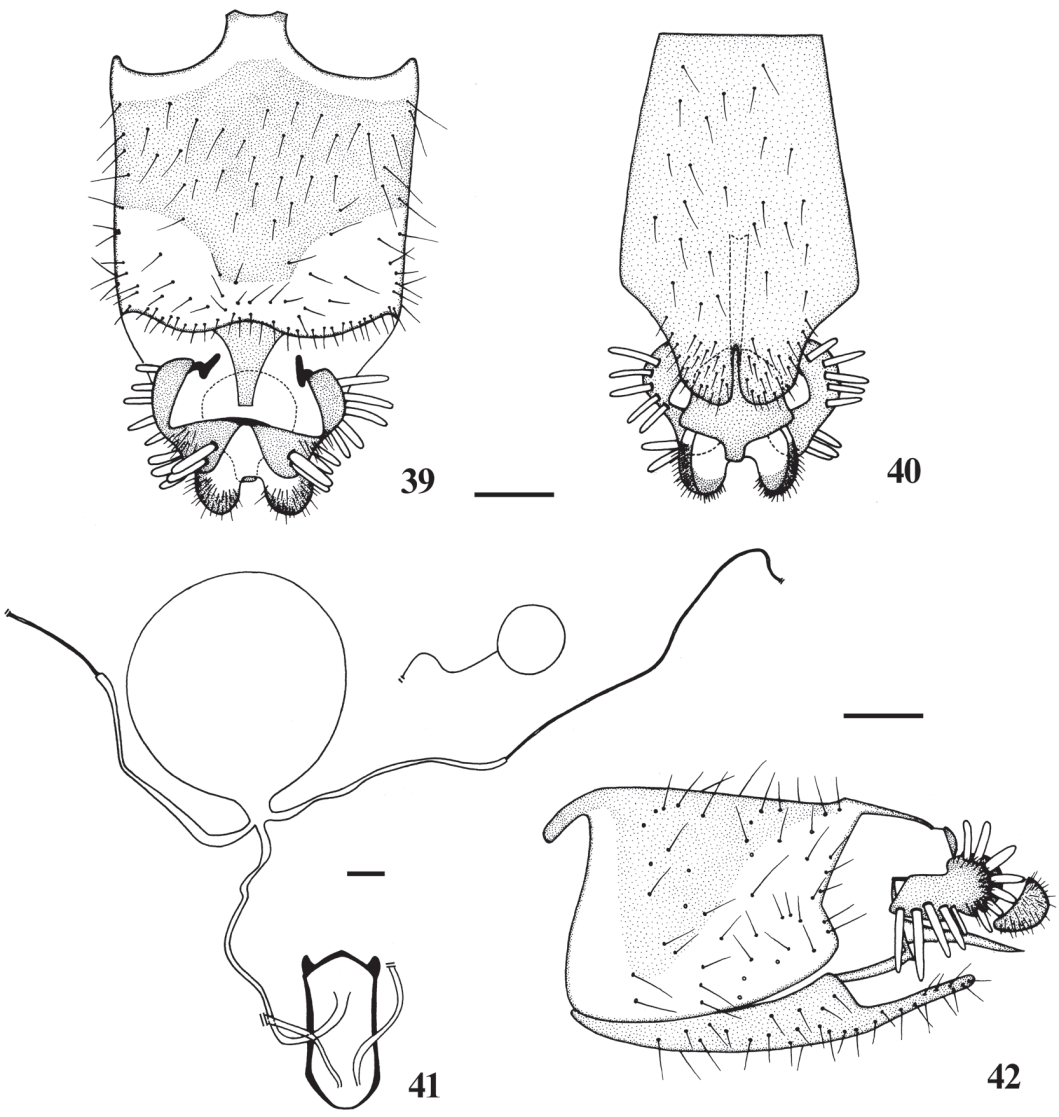
*Dialineura henanensis* Yang. Female. **39** terminalia, dorsal view **40** terminalia, ventral view **41** internal reproductive organs **42** terminalia, lateral view. Scale: 0.2 mm.

### Dialineura nigrofemorata

Kröber, 1937

http://species-id.net/wiki/Dialineura_nigrofemorata

Figs 43 55 73

Dialineura nigrofemorata Kröber, 1937: 272, 290. Type locality: Transbaibalia, Russia (Holotype deposited in Naturhistorisches Museum Wien, Vienna); Zaitzev 1971: 192.
Dialineura intermedia Lyneborg, 1968: 159. Type locality: District SE, Baikal Lake, Russia.

#### Diagnosis.

Male mesonotum with 3 wide black vittae separated by 2 narrow pale grey stripes. Pterostigma of wing yellow. Fore femur only with white pile. Male subepandrial sclerite very long; gonocoxite narrow apically and with substylus in interior margin.

#### Redescription.

Male. Body length 8.2 mm, wing length 7.0 mm.

**Figures 43–47.**
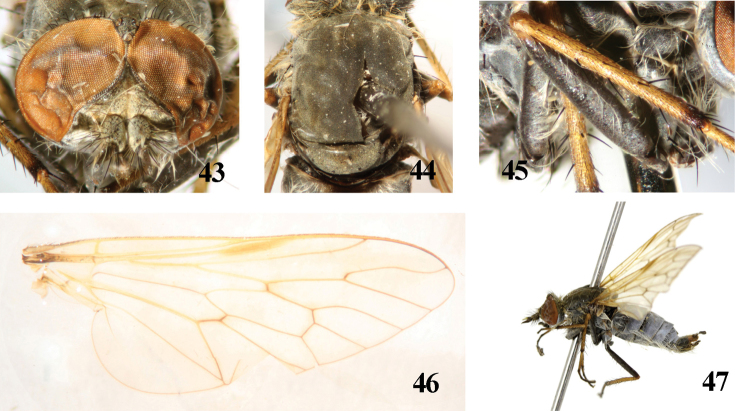
*Dialineura nigrofemorata* Kröber. Male. **43** head, frontal view **44** mesonotum **45** fore femur **46** wing **47** habitus of male, lateral view.

Head (Fig. 43) with dense pale pubescence over black ground color, central upper area of frons brown. White to pale yellow pile from gena to occiput, black setae on ocellar tubercle and frons, setae on frons divided into 2 tufts, parafacial bare, upper occiput also with some black postocular setae. Eyes reddish brown and nearly contiguous on upper frons. Antenna with dense pale pubescence over black ground color, except first flagellomere and style brown; black setae on scape long and thick, but those on pedicel short and thin, first flagellomere nearly bare; central part of first flagellomere widest; style resting apically on first flagellomere with a tiny distal spine; antennal ratio: 3.6 : 1.0 : 3.5 : 0.9. Proboscis black with short brown pile; palpus brown with white pile.

Thorax with dense pubescence over black ground color; mesonotum (Fig. 44) with 3 wide black vittae separated by 2 narrow pale grey stripes. Notum with sparse white pile, prosternum and pleuron with white to pale yellow pile; macrosetae on thorax black. Scutal chaetotaxy (pairs): *np* 3, *sa* 2, *pa* 1, *dc* 1, *sc* 2. Coxae and trochanters pale pollinose over black ground color, femora with pale pubescence over black ground color except apices brownish yellow, tibiae brownish yellow with dark brown apices, all tarsomeres 1 brownish yellow with dark brown apices, other tarsomeres dark brown. White pile present on coxae and femora, setae on legs black. Fore coxa with *a* 1, *av* 1; mid coxa with *a* 3; hind coxa with *a* 3, *d* 1. Fore and mid femora without any prominent setae; hind femur with *av* 7, *pv* 8. Fore tibia with *ad* 4, *pd* 5–6, *pv* 4–6, apically with 6 setae; mid tibia with *ad* 5, *pd* 5, *av* 4, *pv* 4, apically with 7 setae; hind tibia with *ad* 9, *pd* 10–11, *av* 9–10, *pv* 7, apically with 6 setae. Wing (Fig. 46) hyaline tinged yellow; pterostigma very narrow, yellow, at end of R_1_; veins brown except basal surface of wing pale yellow. Halter stalk brownish yellow basally and black apically; knob brown.

Abdomen with dense pubescence over ground color, except tergite 1 and anterior margins of tergites 2–3 with very thin pubescence so that ground color is visible, posterior margin of each segment pale yellow. White pile on abdomen and terminalia. **Male genitalia:** Epandrium (Fig. 51) elongated, 1.3 times longer than wide, apically narrowed with a triangular medial invagination. Subepandrial sclerite slightly constricted in the central area, nearly 3 times longer than cercus. Gonocoxite (Fig. 52) narrow apically and with substylus in interior margin. Distiphallus (Figs 53–55) short and curved, basal part of distiphallus relatively stout.

Female. Unknown.

#### Material. 

1 male, **CHINA:** Liaoning, Xinbin (41°43’N, 125°02’E), 7. VII. 2005, Juan Li.

#### Distribution.

Palaearctic region: China (Liaoning) (Fig. 73), Russia. In China, this is biogeographically part of Northeast Region (Zhang 1999).

#### Remarks.

Kröber (1937) first described a female specimen of *Dialineura nigrofemorata* from Transbaibalia, Russia. Lyneborg (1968) described this species as *Dialineura intermedia* and gave the figures of male genitalia. Zaitzev (1971) redescribed *Dialineura nigrofemorata*, gave the figures of male genitalia and revised *Dialineura intermedia* Lyneborg, 1968 as a synonymy, and he pointed out that the *Dialineura nigrofemorata* described by Lyneborg (1968) in fact was *Dialineura lyneborgi*. We newly record *Dialineura nigrofemorata* from China. This species is similar to *Dialineura lyneborgi* Zaitzev from Russia in the long subepandrial sclerite. But it can be separated from the following features: only white pile present on the fore femur (Fig. 45); the epandrium is nearly 2 times longer than the subepandrial sclerite. In *Dialineura lyneborgi*, black pile distinctly present on fore femur; the epandrium is 1.5 times longer than the subepandrial sclerite (Zaitzev 1971).

**Figures 48–55.**
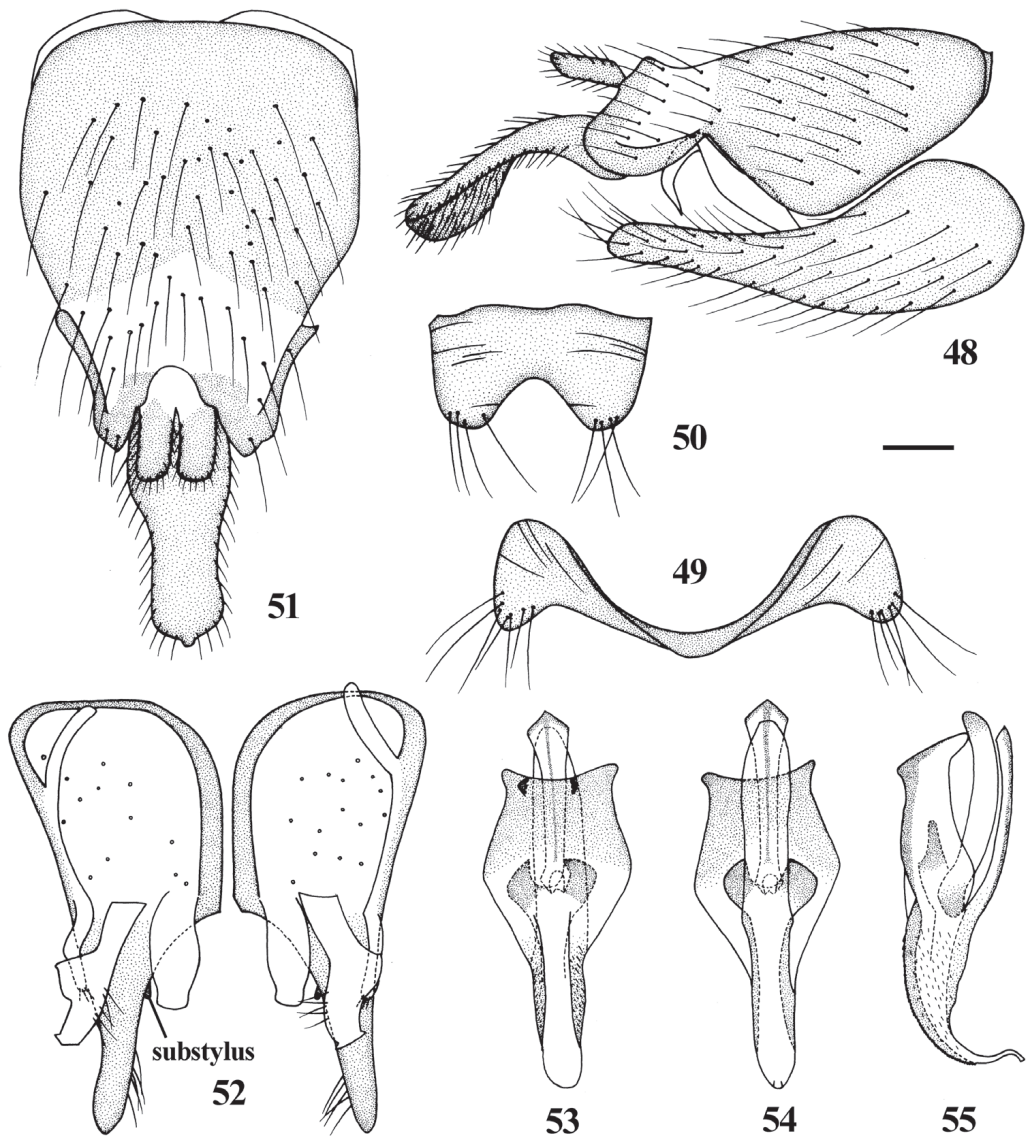
*Dialineura nigrofemorata* Kröber. Male. **48** terminalia, lateral view **49** tergite 8 **50** sternite 8 **51** epandrium, cercus and subepandrial sclerite, dorsal view **52** gonocoxite and gonostylus, dorsal view **53** aedeagus, dorsal view **54** aedeagus, ventral view **55** aedeagus, lateral view. Scale: 0.2 mm.

### Dialineura gorodkovi

Zaitzev, 1971

http://species-id.net/wiki/Dialineura_gorodkovi

Figs 56, 57 73

Dialineura gorodkovi Zaitzev, 1971: 191. Type locality: Chukchi, Russia (Holotype deposited in Zoological Institute, Academy of Science of Russia, St. Petersburg); Lyneborg 1975: 577; Webb and Irwin 1991: 873.

#### Diagnosis.

**Figures 56, 57.**
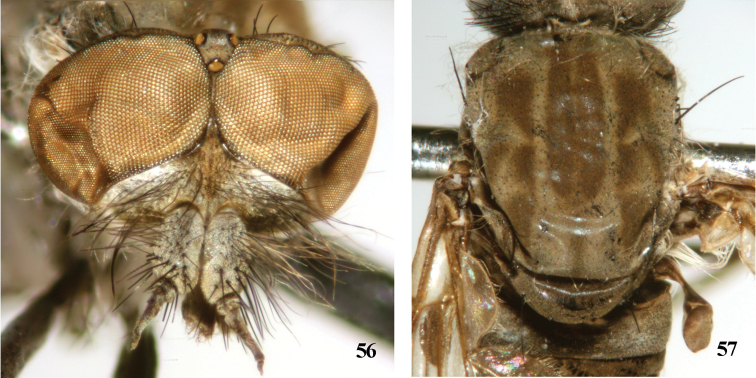
*Dialineura gorodkovi* Zaitzev. Male. **56** head, frontal view **57** mesonotum.

Black setae on frons (Fig. 56) very long and dense, even expand to parafacicals. Male mesonotum (Fig. 57) with 3 wide dark brown vittae, separated by 2 narrow brownish grey stripes, the central vitta with a narrow brownish grey stripe in the middle. Pterostigma of wing dark brown. Male subepandrial sclertie (Zaitzev 1971, p191, fig. 5; Webb and Irwin 1991, p872, fig. 5) triangular and 1.5–2 times longer than cercus; gonocoxite (Zaitzev 1971, p191, fig. 5; Webb and Irwin 1991, p872, fig. 7, 8) narrow apically and with substylus in interior margin.

#### Materials.

2 male, **CHINA:** Beijing, Xiaolongmen (39°57’N, 115°26’E), 21. V. 2010, Tao Li.

#### Distribution.

Palaearctic region: China (Beijing) (Fig. 73), Russia; Nearctic region: Canada and USA. In China, this is biogeographically part of North China Region (Zhang 1999).

#### Remarks.

Zaitzev (1971) firstly described *Dialineura gorodkovi* from Chukchi, Russia and gave the figures of the male genitalia. Lyneborg (1975) first recorded D. gorodkovi in north America. Webb and Irwin (1991) redescribed *Dialineura gorodkovi* and gave figures of both male and female genitalia. We newly record *Dialineura gorodkovi* from China.

### Dialineura affinis

Lyneborg, 1968

http://species-id.net/wiki/Dialineura_affinis

Figs 58 69 73

Dialineura affinis Lyneborg, 1968: 157. Type locality: Sichuan, China (Holotype deposited in U. S. National Museum, Washington D. C.); Zaitzev 1971: 187; Yang 1999: 186.

#### Diagnosis.

Frons with white pile. Pterostigma of wing brownish yellow. Male gonocoxite narrow apically and with substylus in interior margin; distiphallus serrated at lateral edges.

#### Redescription.

Male. Body length 8.1–8.5 mm, wing length 6.0–7.2 mm.

Head (Fig. 59) with dense pale pubescence over black ground color. White pile on frons and from gena to occiput, brown pile on ocellar tubercle, parafacial bare, upper occiput also with some black postocular setae. Eyes reddish brown and nearly contiguous on upper frons. Antenna with dense pale pubescence over black ground color; black setae on scape long and thick, but those on pedicel short and thin, scape also covered with long white pile, first flagellomere nearly bare; central part of first flagellomere widest; style resting apically on first flagellomere with a tiny distal spine; antennal ratio: 5.3 : 1.0 : 4.3 : 0.8. Proboscis brownish yellow with short brown pile; palpus pale brownish yellow with white pile.

Thorax with dense pale pubescence over black ground color (as the pubescence on the mesonotum of specimens are totally scraped off, the pattern on the mesonotum is unknown). Notum with dense white pile, prosternum and pleuron with dense white pile; macrosetae on thorax black. Scutal chaetotaxy (pairs): *np* 3, *sa* 2, *pa* 1–2, *dc* 2, *sc* 2. Coxae and trochanters pale pollinose over black ground color, femora with pale pubescence over black ground color except apices brownish yellow, tibiae brownish yellow with dark brown apices, all tarsomeres 1 brownish yellow with dark brown apices, other tarsomeres dark brown. White pile present on coxae and femora, setae on legs black. Fore coxa with *a* 1, *av* 1; mid coxa with *a* 3; hind coxa with *a* 2, *d* 1. Fore and mid femora without any prominent setae; hind femur with *av* 6, *pv* 3. Fore tibia with *ad* 3–4, *pd* 3, *pv* 4, apically with 4–5 setae; mid tibia with *ad* 3–4, *pd* 4, *av* 5, *pv* 4, apically with 6 setae; hind tibia with *ad* 7–9, *pd* 8, *av* 7–8, *pv* 6–7, apically with 5 setae. Wing (Fig. 60) hyaline tinged yellow; pterostigma very narrow, brownish yellow, at end of R_1_; veins brown except upper basal surface of wing yellow. Halter stalk yellow but dark brown apically; knob brown.

Abdomen with dense pale pubescence over black ground color, except tergite 1 and terminalia with very thin pubescence so that ground color is visible, posterior margin of each segment pale yellow. White pile on abdomen and terminalia. **Male genitalia:** Epandrium (Fig. 65) elongated, 1.5 times longer than wide, apically narrowed with a triangular medial invagination. Subepandrial sclerite trapezoid, little longer than cercus. Gonocoxite (Fig. 66) with substylus, relatively narrow apically. Distiphallus (Figs 67–69) short and curved, serrated at lateral edges.

Female. Unknown.

#### Materials.

3 male, **CHINA:** Tianjin, Qingguang Farm (39°13'N, 117°00'E), 9. IV. 1965; 1 male, **CHINA:** Tianjin, Qingguang Farm (39°13'N, 117°00'E), 10. IV. 1965. The collectors are totally unknown.

#### Distribution.

Oriental region: China (Sichuan); Palaearctic region: China (Tianjin) (Fig. 73). This is biogeographically part of Southwest Region and North China Region (Zhang 1999).

#### Remarks.

Lyneborg (1968) first described *Dialineura affinis* from Sichuan, China as a very special species because of “The face bears long blackish hairs similar to those on the frons”, and he gave the figures of male genitalia. Irwin and Lyneborg (1981a) pointed it out again - “Lateral portion of face usually bare, only pilose in *affinis* Lyneborg from China” – as an exception of the general characters of *Dialineura*. Zaitzev (1971) recorded it in the revision of Palaearctic species of the genus *Dialineura*. Yang (1999) included it into the key to species of *Dialineura* from China. We find the structures of male genitalia of our materials are almost same as *Dialineura affinis*, especially in the distiphallus with the serrated lateral edges; therefore we identify our materials as *Dialineura affinis*. However, the parafacial (Fig. 61) of our specimens are totally bare without any pile or setae, if the “face” (Lyneborg 1968) and the “lateral portion of face” (Irwin and Lyneborg 1981a) refer to the same structure of parafacial. Moreover, the frons (Fig. 56) of our specimens are covered with only white pile instead of blackish setae.

**Figures 58–61.**
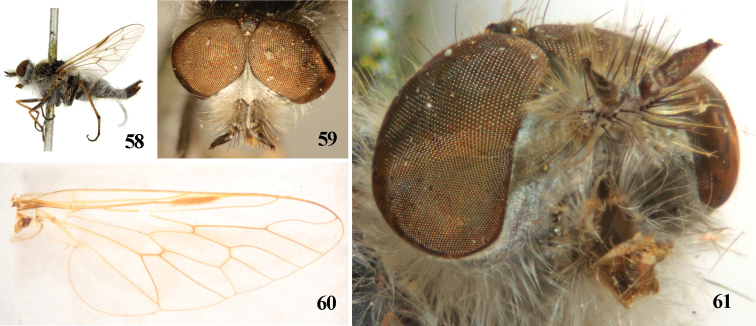
*Dialineura affinis* Lyneborg. Male. **58** habitus of male, lateral view **59** head, frontal view **60** wing **61** parafacial.

**Figures 62–69.**
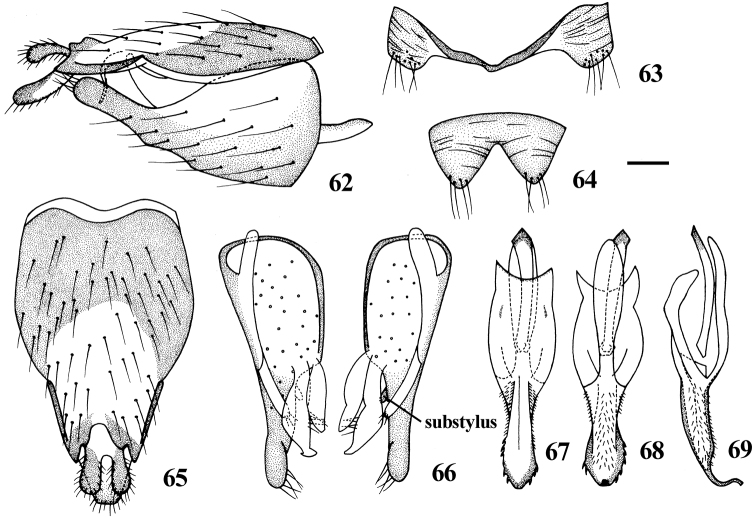
*Dialineura affinis* Lyneborg. Male. **62** terminalia, lateral view **63** tergite 8 **64** sternite 8 **65** epandrium, cercus and subepandrial sclerite, dorsal view **66** gonocoxite and gonostylus, dorsal view **67** aedeagus, dorsal view **68** aedeagus, ventral view **69** aedeagus, lateral view. Scale: 0.2 mm.

### Dialineura kikowensis

Ôuchi, 1943

http://species-id.net/wiki/Dialineura_kikowensis

Figs 70, 71 73

Dialineura kikowensis Ôuchi, 1943: 481. Type locality: Zhejiang, China (Holotype deposited in Shanghai Entomological Museum, Shanghai); Yang 1999: 186.

#### Diagnosis. 

Female mesonotum with two wide yellow vittae. Apical margin of cell m_3_ narrower than cross-vein m-cu (Ôuchi 1943, p481, fig. 1). All femora (Fig. 67) entirely yellow. Each tergite of abdomen with a large black central spot.

#### Material.

Holotype female, **CHINA:** Zhejiang, Xikou (29°41’N, 121°16’E), 11. V. 1936. The collector is unknown.

#### Distribution.

Oriental region: China (Zhejiang) (Fig. 73). This is biogeographically part of Central China Region (Zhang 1999).

#### Remarks.

Ôuchi (1943) described only one female specimen of *Dialineura kikowensis* from Zhejiang, China and gave the figures of middle part of wing and abdomen in dorsal view. Yang (1999) included it into the key to species of *Dialineura* from China. We examine photos of the type specimen.

**Figures 70, 71.**
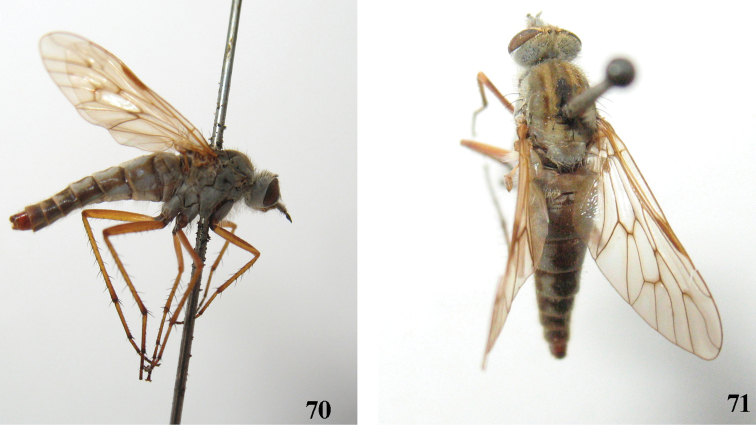
*Dialineura kikowensis* Ôuchi. Female. **70** habitus of female, lateral view **71** habitus of female, dorsal view.

### Dialineura aurata

Zaitzev, 1971

http://species-id.net/wiki/Dialineura_aurata

Figs 72 73

Dialineura aurata Zaitzev, 1971: 198. Type locality: Southern seaside of Russia (Holotype deposited in Zoological Institute, Academy of Science of Russia, St. Petersburg); Zaitzev 1977: 128; Yang 1999: 186.

#### Diagnosis.

Head and entire body covered with dense bright yellow pubescence. Legs pale yellow. Segment 8 of abdomen shinny black. (Zaitzev 1971)

#### Distribution.

Palaearctic region: China (Northeast region) (Fig. 73) (Zhang 1999), Russia.

#### Remarks.

Zaitzev (1971) described four female specimens of *Dialineura aurata* from Palaearctic region including one from Northeast China. Zaitzev (1977) newly recorded three female specimens of *Dialineura aurata* from the Far East region of Russia. Yang (1999) included it into the key to species of *Dialineura* from China.

**Figure 72.**
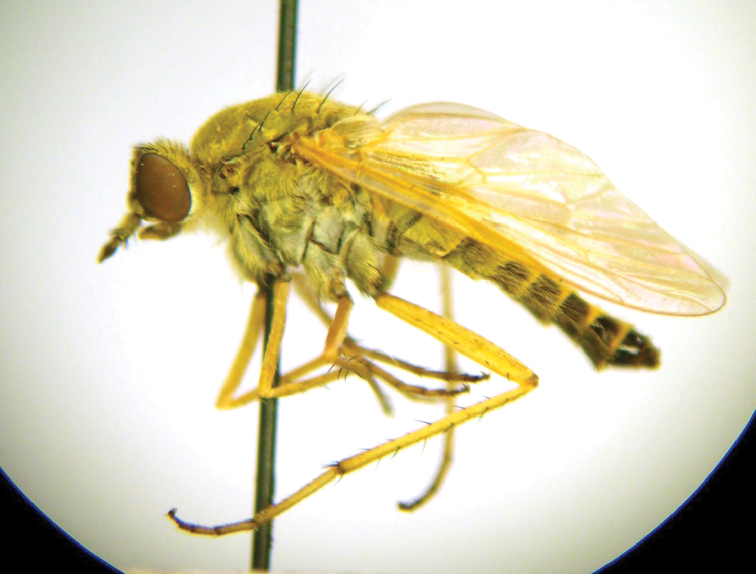
Habitus of female *Dialineura aurata* Zaitzev, 1971, lateral view.

**Figure 73.**
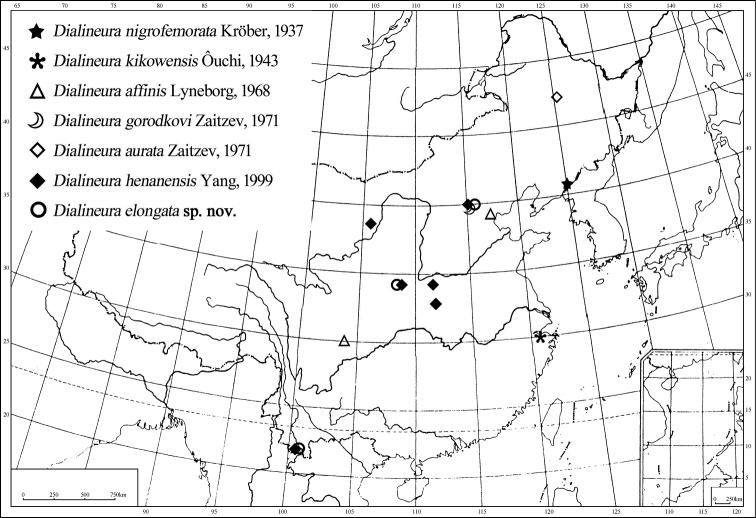
Distribution of *Dialineura* in China.

## Supplementary Material

XML Treatment for Dialineura elongata

XML Treatment for Dialineura henanensis

XML Treatment for Dialineura nigrofemorata

XML Treatment for Dialineura gorodkovi

XML Treatment for Dialineura affinis

XML Treatment for Dialineura kikowensis

XML Treatment for Dialineura aurata
